# Evaluating the protective potency of *Acacia hydaspica* R. Parker on histological and biochemical changes induced by Cisplatin in the cardiac tissue of rats

**DOI:** 10.1186/s12906-019-2575-8

**Published:** 2019-07-23

**Authors:** Tayyaba Afsar, Suhail Razak, Ali Almajwal, Maria Shabbir, Muhammad Rashid Khan

**Affiliations:** 1 Department of Biochemistry, Faculty of Biological Sciences, Quaid-i-Azam University, Islamabad, Pakistan; 2 Department of Animal Sciences, Faculty of Biological Sciences, Quaid-i-Azam University, Islamabad, Pakistan; 3 Department of Community Health Sciences, College of Applied Medical Sciences, King Saud University, Riyadh, Saudi Arabia; 4 Atta-ur-Rahman School of Applied Biosciences, NUST, Islamabad, Pakistan

**Keywords:** Cisplatin, Cardiotoxicity, Oxidative trauma, Cardiac function biomarkers, Antioxidant enzymes, Morphological alterations

## Abstract

**Background:**

Increase oxidative trauma is the main cause behind Cisplatin (CP) induced cardiotoxicity which restricts its clinical application as anti-neoplastic prescription. *Acacia hydaspica* is a natural shrub with diverse bioactivities. *Acacia hydaspica* ethyl acetate extract (AHE) ameliorated drug-induced cardiotoxicity in animals with anti-oxidative mechanisms. Current study aimed to evaluate the protective potential of *A. hydaspica* against cisplatin-induced myocardial injury.

**Methods:**

Rats were indiscriminately distributed into six groups (*n* = 6). Group 1: control; Groups 2: Injected with CP (7.5 mg/kg bw, i.p, single dose) on day 16; Group 3: Treated for 21 days with AHE (400 mg/kg b.w, oral); Group 4: Received CP injection on day 16 and treated with AHE for 5 days post injection; Group 5: Received AHE (400 mg/kg b.w/day, p.o.) for 21 days and CP (7.5 mg/kg b.w., i.p.) on day 16; Group 6: Treated with silymarin (100 mg/kg b.w., p.o.) after 1 day interval for 21 days and CP injection (7.5 mg/kg b.w., i.p.) on day 16. On 22nd day, the animals were sacrificed and their heart tissues were removed. Cisplatin induced cardiac toxicity and the influence of AHE were evaluated by examination of serum cardiac function markers, cardiac tissue antioxidant enzymes, oxidative stress markers and histology.

**Results:**

CP inoculation considerably altered cardiac function biomarkers in serum and diminished the antioxidant enzymes levels, while increased oxidative stress biomarkers in cardiac tissues AHE treatment attenuated CP-induced deteriorations in creatine kinase (CK), Creatine kinase isoenzymes MB (CK-MB), cardiac Troponin I (cTNI) and lactate dehydrogenase (LDH) levels and ameliorated cardiac oxidative stress markers as evidenced by decreasing lipid peroxidation, H_2_O_2_ and NO content along with augmentation in phase I and phase II antioxidant enzymes. Additionally, CP inoculation also induced morphological alterations which were ameliorated by AHE. In pretreatment group more significant protection was observed compared to post-treatment group indicating preventive potential of AHE. The protective potency of AHE was comparable to silymarin.

**Conclusion:**

Results demonstrate that AHE attenuated CP induce cardiotoxicity. The polyphenolic metabolites and antioxidant properties of AHE might be responsible for its protective influence.

## Background

Clinical implication of Cisplatin (*cis*-dichlorodiammine-platinum (II), CP) is restricted owing to augmented oxidative stress and apoptosis that have been involved in cardiac injury. Cisplatin usage is also associated with the cardiotoxicity including arrhythmia, myocarditis, cardiomyopathy and congestive heart failure [1–5]. CP treatment in combination with complementary anticancer medications such as methotrexate, 5-fluorouracil, bleomycin and doxorubicin is concomitant with fatal cardiac disorders [6]. CP-containing chemotherapy to the patients encourages deterioration in the abundance of various antioxidants in the plasma, which may lead to the failure of anti-oxidative defense mechanism. Besides, the genotoxicity of cisplatin may result in the inception of tributary afflictions in healthy tissues [7, 8]. CP induces lacerations in the vascular components of the cardiac tissues of treated animals. The inimical effects comprised of congestion, perivascular edema, hemorrhage, expanded medial muscle thickness, hyalinization with the constrained luminal area in the coronary and intramuscular arterioles. The vascular endothelium perform various tasks and CP consequent endothelial deteriorations are accountable for several health complications, such as atherosclerosis, high blood pressure, thrombosis, vasculitis, sepsis and bleeding etc. [9].

In current scenario, the leading objective of scientific exploration concentrating on platinum complexes is to ascertain compounds that have greater potency, minimum toxicity, curtail cross-resistance and enriched pharmacological features as compared with the parent drug; CP [10, 11]. There are cumulative facts proposed that the consumption of antioxidants could be effective in impeding cisplatin-prompted toxicity [12–14]. Medicinal plants and natural herbal ingredients have promising antioxidant potency and are gaining importance in controlling several ailments [15]. Silymarin is a flavonoid isolated from *Silybum marianum,* has previously been successfully utilized as a remedial agent in numerous clinical and in vivo and in vitro experimental models of liver toxicity [16], nephrotoxicity [17] and cardiotoxicity [5]. Silymarin being an antioxidant flavonoid complex holds the capability to quench free radicals, chelate metal ions, alleviate the membrane permeability via impeding lipid peroxidation and precluding glutathione depletion [16, 18, 19]. Silymarin (100 mg/kg, orally, for 10 days) secure cardiac tissue against the cisplatin-prompted damage by decreasing the action of serum biochemical markers comprising lactate dehydro genase (LDH), creatine kinase isoenzyme MB (CK-MB) and cardiac troponin I (cTnI) by its anti-lipid peroxidation action. Consequently, silymarin brought equilibrium of cardiac membranes and precluded the seepage of cardiac enzymes. Additionally, silymarin precise the lipid peroxidation due to the occurrence of free hydroxyl groups at C5 and C7 carbons, which counter with peroxy radicals. Silymarin increased the actions of endogenous antioxidant enzymes such as SOD and lessened oxidative mitochondrial DNA damage due to the free radical scavenging property [20].

*Acacia s*pecies are a great source of polyphenolic metabolites, acknowledged to have substantial antioxidant properties that benefit in the deterrence against several oxidative traumas related diseases comprising cardiovascular, neurodegenerative and cancer [21–23]. *Acacia hydaspica* R. Parker; synonym *A. eburnea* stood in family Leguminosae [24], owns antioxidant, anticancer, anti-hemolytic [25], anti-inflammatory, antipyretic, analgesic [26], antidepressant [27] and hepato-protective and protective against testicular toxicity [28, 29]. Bioactive metabolites identified in *Acacia* are i.e., gallic acid, catechin, rutin, caffeic acid, 7-*O*-galloyl catechin, +catechin and methyl gallate [26, 30]. Previous researches indicated that Catechin significantly reduce idarubicin-induced cardiotoxicity in rats. Catechin also exhibit cardioprotective effect in Dox-treated animals [31].Catechin ameliorated electrocardiogram (ECG) fluctuations and myocardial contractility [32]. *Acacia* species were reported to possess cardioprotective potential in animal models. *A. Senegal* gum Arabic displayed a defensive influence against doxorubicin-persuaded cardiac insult by attenuating cardiomyocyte injuries and ameliorating altered serum cardiac function biomarkers [33]. Another study in rabbits indicated that *A. senegal* seed extract administration ameliorated atherogenic diet induced cardiac LPO and histopathological abnormalities in aorta wall, heart and kidney. *Acacia hydaspica* ethyl-acetate extract (AHE) showed the cardioprotective effect against doxorubicin induced oxidative stress. AHE treatment prevents structural cardiomyocytes degeneration via inhibiting the oxidative stress marker levels, decreasing CK, CK-MB and LDH, and increasing cardiac antioxidant status [34]. Ethyl acetate extract of *A. hydaspica* (AHE) showed protection against cisplatin induced reproductive and hepatic toxicity in rats by improving endogenous antioxidant defense [28, 29].

Centered on prior explorations on the cardio-protective prospective of the *Acacia* species, polyphenolic composites in animal models and protective potential of *A. hydaspica*; current investigation was designed to investigate the effect of ethyl-acetate extract of *A. hydaspica* in comparison with silymarin against CP-induced cardiac toxicity and oxidative stress in rats. Silymarin was selected as a standard herbal extract with proven beneficial effects. Antioxidant enzymes, oxidative stress biomarkers and biochemical serum cardiac function markers levels were determined along with histopathological examination to evaluate the efficiency of *A. hydaspica* against CP- persuaded cardiac injuries.

## Methods

### Collection of plant

*A. hydaspica (*Aerial parts) were obtained from Kirpa charah region Islamabad, Pakistan. Plant specimen was recognized by Dr. Sumaira Sahreen (Curator at Herbarium of Pakistan, Museum of Natural History, Islamabad) and deposited (Accession No. 0642531) at the herbarium of Pakistan, museum of natural history, Islamabad.

### Preparation of extract

The aerial parts (twigs and leaves) of the plant were shade dried. Dried plant was processed in electrical grinder to obtain fine powder. The methanol extract was obtained by allowing 3 kg of powder to macerate 3 times in 95% methanol (3 × 4000 ml) for 5 consecutive days. The supernatant was filtered and filtrate was evaporated by rotary vacuum evaporator (Buchi, R114, Switzerland) to obtain a viscous mass as the crude methanol extract (AHM). 12 g of AHM was suspended in water (250 ml) with continuous stirring then successively added (3 × 200 ml) following solvents; *n*-hexane, ethyl acetate, chloroform and *n*-butanol respectively, and each layer was allowed to separate for 3 h in a separating funnel and at last water soluble fraction (AHA) was obtained. All fractions were dried using rotary evaporator. *Acacia hydaspica* crude methanol extract (AHM) yield was 15% of the dry powder, while AHH, AHE, AHC, AHB and AHA yielded 5.27, 27.77, 1.94, 41.66 and 8.05% respectively, of dry methanol extract. Ethyl acetate extract (AHE) (the most bioactive extract under in vitro examinations and containing bioactive polyphenols [35] was selected for further in vivo investigation. Table 1 indicated the phytochemical composition of AHE and bioactivities of metabolites.

**Table 1:** Phytochemical composition of *Acacia hydaspica* ethyl acetate extract (AHE)

Detection method	Phytoconstituents	References	Biological activity/Chemopreventive potential /Refrences
Qualitative screening	Tannin	[25]	Antioxidant, anticancer [36], Nehroprotective against CP, increase bioavailability of CP [37], inhibit CP–induced TBARS production in rat kidney [38].
	Steroids		Anti-inflammatory [39, 40], anticancer [41], Nehroprotective against CP [42]
	Alkaloids		Anti-inflammatory [39, 40], antioxidant, chemopreventive, anticancer [43], nephroprotective against CP-induced renal injury via inhibition of oxidative/nitrosative stress, inflammation, autophagy and apoptosis [44].
	Flavonoids		Anti-inflammatory [39, 40, 45], antioxidant, anticancer, ameliorate cisplatin-induced nephrotoxicity via anti-apoptotic and anti-inflammatory effects [46].
	Coumarins		Anti-inflammatory [47], antioxidant [48], anti-cancer [49].
	Terpenoids		Antiinflammatory [5, 6], chemopreventive, anticancer [10] renoprotective against CP [11].
Quantitative Estimation	*Flavonoids*: 129 ± 1.32 TFC (mg rutin equivalent/g dry sample)	[25]	Antioxidant, anticancer, induced apoptosis, inhibit oxidative stress, flavonids exibit protection against CP induced nephrotoxicity [36], i.e. rutin a flavonoid effectively reduced the cisplatin-induced renal toxicity in albino rats by ameliorating serum kidney function markers, creatinine clearance, and renal malondialdehyde levels [50].
	*Phenolics*: 120.3 ± 1.15 TPC (mg gallic acid equivalent/g dry sample)		Antioxidant,anticancer [51], anti-inflammatory, antitumour, anti-proliferative [52], chemopreventive and anticancer activity of GSE in various cancers attribitued to the presence of polyphenolics constituents and their antioxidant potential [53].
HPLC-DAD Screening using standard flavonoids	Gallic acid (4.52 52.92 μg/100 mg dry powder)	[25]	Antioxidant, antimutagenic, chemopreventive [54], inhibit CP induce acculmalation of TBARS in renal tissues in vitro [38], modulate antioxidant status and prevent CP induce kidney demage in rats [55].
	Catechin (11.438648.0 μg/100 mg dry powder)		Antioxidant, anticancer [56], chemopreventive [57]
	Myricetin (17.08 34.60 μg/100 mg dry powder)		Myricetin exhibited a protective against cisplatin-induced nephrotoxicity in rats due to its antioxidant and anti-inflammatory effects [58] phenolic compounds present in extracts justify their marked antioxidant activities.
Isolation of pure bioactive compounds through bioassay guided fractionation and isolation.	7-*O*-galloylcatechin (187.5 mg/g)	[30, 45]	Antioxidant [59], anticancer, antiproliferative and apoptotic against breast and prostate cancer [30], prevented CP-induced oxidative stress, inflammation, and apoptosis [60].
	+Catechin (100 mg/g)		Anticancer, antioxidant, proapoptotic [30], nephroprotective; renoprotective effect of catechin hydrate against gentamicin-induced nephrotoxicity might be mediated through its antioxidant and possible direct nephroprotective actions [61].
	Methyl gallate (37.5 mg/g)		Anticancer, antioxidant [30, 62], prevent oxidative stress and DNA damage in renal cells via scavenging of intracellular reactive oxygen species (ROS), inhibition of lipid peroxidation and prevention of intracellular GSH depletion [63].

*TFC*: Total flavonoid content, *TPC*: Total phenolic content. Phytoconstituents of *Acacia hydaspica* ethylacetate fractions derived from previous investigations

### Acute toxicity evaluation

The acute toxicity study was conducted as per the guidelines 425 of Organization for Economic Cooperation and Development (OECD) for testing of chemicals for acute oral toxicity [64]. The thorough method is described in our earlier study [28]. General behavioral fluctuations were noticed by the hitherto procedure [65]. Animals were observed constantly for 2 h for any sort of convulsion, tremor, aggression, excitation, loss of grasp, different reactivity to touch, and sedation [66]. AHE was proved to be harmless at all tested quantities (up to 4000 mg/kg b.w) and it did not convinced any noxious sign in rats like sedation, convulsions, diarrhea and irritation.

### Preparation of dose for treatment

Cisplatin (CP) injection (Sigma-Aldrich, St. Louis, MO, U.S.A.) was diluted in saline to make precise amount (7.5 mg/kg body weight) for administration [67]. Silymarin (100 mg/kg b.w) and AHE (400 mg/kg b.w) were freshly prepared in distilled water prior to every administration. 400 mg/kg b.w. dose of AHE and 100 mg mg/kg b.w. of silymarin was selected based on our pilot experiment. AHE and Silymarin are given in volume of 2 ml in feeding tubes.

### Scheme of experiment

Sprague Dawley rats (200–225 g) were obtained from the Primate Facility at Quaid-i-Azam University, Islamabad. The animals were kept in routine cages at room temperature under maintined12 h light/dark cycle, fed with normal pellet diet and tap water. Procedures of national institute of animal health (NIH guidelines) were strictly followed for conducting the experimental procedures. The ethical board of Quaid-i-Azam University, Islamabad permitted the investigational procedure (Bch#264). Animals were allocated into six groups (*n* = 6). Sample size is selected based on “resource equation” method [68]. As ANOVA test was used to compare significant differences in all treatment groups. If more number of animals selected then it may lead to unnecessary wastage of resources and may lead to ethical issues. The treatment procedure was adopted according to previous studies [69, 70] with slight adjustments.

Group 1: Control; given distilled water by feeding tube (2 ml)

Group 2: CP treated; received one dose of CP (7.5 mg/kg b.w., i.p.) on day 16th of experiment.

Group 3: AHE treated; 400 mg/kg body weight/day oral dose for 21 days

Group 4: CP + AHE (post-treated group); CP on day 16 and AHE (400 mg/kg b.w/day, p.o.) was given from day 16 to 21.

Group 5: AHE + CP (pre-treated group); received 400 mg/kg body weight/day, p.o. for 21 days and CP (7.5 mg/kg b.w., i.p.) on day 16.

Group 6: Silymarin+CP; received 100 mg/kg b.w., p.o. dose every other day (11 doses/21 days) and CP (7.5 mg/kg b.w., i.p.) on day 16.

Body weights of rats were noted at the start and completion of experiment. Animals were sacrificed by decapitation. Heart was removed and washed with ice cold saline and dried with blotting paper before weighted. Next, part of the organs were treated with liquid nitrogen and stored at − 80 °C for further enzymatic analysis while the remaining portion was stored in 10% phosphate buffered formalin for histological examination.

### Biochemical investigations

#### Cardiac function biomarkers

Biomarker enzymes for cardiac function viz.; CK, CK-MB, LDH and cholesterol were examined following the protocol of AMP diagnostic kits (Stattogger Strasse 31b 8045 Graz, Austria). Cardiac Troponin I (cTnI) levels in the plasma obtained from the animals were measured by enzyme-linked fluorescent assay using the VIDAS Troponin I Ultra kit following manufacturer protocol.

#### Homogenate preparation

Tissues sample (100 mg) was homogenized in 10 volume of 100 mM KH_2_PO_4_ buffer containing 1 mM EDTA, pH 7.4. The homogenate was centrifuged at 12000×g for 30 min at 4 °C to obtain the supernatant which was then stored at − 20 °C for enzymes assays.

#### Measurement of protein content

Total soluble proteins within the cardiac tissues were estimated by earlier protocol [71]. Bovine serum albumin (BSA) standard calibration curve was used to evaluate amount of serum proteins in the sample.

##### *Enzymatic antioxidant* analysis

CAT activity was calculated by the method of Tayyaba et al. with minor changes. Kakkar et al. method was exploited for the determination of superoxide dismutase (SOD) and Quinone reductase (QR) amount in the cardiac tissues of various treatment groups [72]. Reduced glutathione (GSH) action was tested as established by Jollow [73]. Plan of Habig et al. [74] was pursued for the valuation of GST potency. Glutathione reductase action in tissue samples was examined as described by Carlberg and Mannervik [75]. Glutathione peroxidase assay (GPx) was conducted as performed by Mohandas and coworkers [76]. The activity of γ-glutamyl transpeptidase (γ-GT) was tested using Orlowski et al. method [77].

#### Measurement of oxidative stress markers

Procedure of Iqbal et al. [78] was implemented with trivial revisions for the valuation of lipid peroxidation (TBARs). Approximation of hydrogen peroxide levels in tissue samples was examined by technique described formerly [79]. For the accomplishment of nitrite assay, Griess reagent was used [80].

##### Histopathological analysis

For histopathological inspection, cardiac tissues sections from each group were placed in a fixative comprising absolute alcohol (85 ml), glacial acetic acid (5 ml) and 40% formaldehyde (10 ml). After dehydration tissue samples were secure in paraffin to prepare blocks for microtomy. 4–5 μm sections of tissues were cut with microtome and stained with Hemotoxilin-Eosin (H&E) and examined for morphological alterations under light microscope (DIALUX 20 EB, at 40X).

#### Statistical check

Data are presented as mean ± SEM (*n* = 6). Statistical differences between different treatments were calculated by one way analysis of variance (ANOVA) followed by Tukey’s test on Graph pad prism 5software. Significance level was set at *p* < 0.05.

## Results

### Effect on heart and body weight

CP treatment showed insignificant difference in heart weight/body weight (HW/BW) ratio compared to control rats, while noteworthy reduction in body weight was noticed in CP alone or in CP + AHE groups compared to control rats. Significant protection was observed in AHE+ CP and Sily + CP groups as against the CP group (Table 2). No significant deviations in the BW gain or HW/BW ratio were observed in rats treated with AHE alone. No deaths were seen in any of treatment groups.

**Table 2:** Effect of DOX and/or AHE treatment on body weight, heart weight and heart /body weight ratio of rats

Treatment (mg/kg)	Body weight (BW)(g)		Heart weight (HW)(g)	Ratio (HW/BWx10^3^)
	Initial	Final		
Control	218.0 ± 1.065	259.7 ± 1.202	0.692 ± 0.03	2.66
CP	220.0 ± 1.56	226.0 ± 1.033^***^	0.502 ± 0.034^**^	2.22
AHE alone	219.5 ± 1.02	260.0 ± 1.07^+++^	0.699 ± 0.025^+++^	2.68
CP + AHE	220.5 ± 1.06	244.0 ± 1.15^***,+++^	0.592 ± 0.013^*,#^	2.42
AHE + CP	220.3 ± 0.882	256.3 ± 1.14^+++,###^	0.690 ± 0.024^b**^	2.69
Sily + CP	222.0 ± 0.577	256.5 ± 922^b^	0.691 ± 0.019^b**^	2.69

Data are represented mean ± S.E.M (*n* = 6). Asterisks ^*, **, ***^ indicates *p* < 0.05, 0.001, 0.0001 vs. control; ^+, ++, +++^ indicates *p* < 0.05, 0.001, 0.0001 vs. CP alone; ^#, ##, ###^ indicates *p* < 0.05, 0.001, 0.0001 vs. AHE + CP. Non-significant difference (*p* > 0.05) was recorded between control and AHE alone treated group in all parameters. (One way ANOVA followed by Tukey’s multiple comparison tests). Sily-Silymarin

### Influence of AHE on serum cardiotoxicity biomarkers

**Fig. 1:**
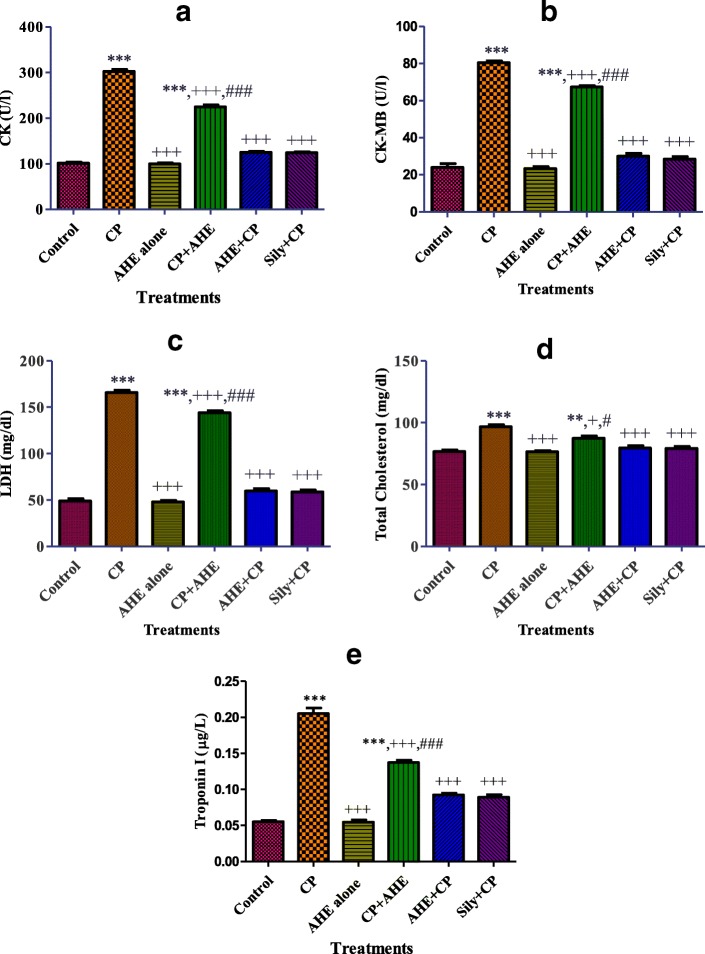
Effect of AHE treatment on serum markers of cardiac injury. **a**: CK, **b**: CK-MB, **c**: LDH, **d**: cholesterol and **e**: Troponin I. Data are represented mean ± S.E.M (*n* = 6). Asterisks ^*, **, ***^ indicates *p* < 0.05, 0.001, 0.0001 vs. control; ^+, ++, +++^ indicates *p* < 0.05, 0.001, 0.0001 vs. CP; ^#, ##, ###^ indicates *p* < 0.05, 0.001, 0.0001 vs. AHE+ CP

Acute administration of CP (7.5 mg/kg b.w) induced cardiotoxicity and exhibited noteworthy (*p* < 0.0001) increase in serum cardiac biomarkers viz. CK, CK-MB, LDH, cholesterol and cTnI, in contrast to the control group (Fig. 1a, b, c, d and e respectively). The elevated level of cardiac function biomarkers especially cTnI, CK and CK-MB are well recognized quantitative index of cardiac tissue damage. The CP-induced elevations in cardiac tissue damage biomarkers were ameliorated (*p* < 0.0001) with AHE treatments, both either before or after toxicity, and the effect was extra prominent in case of AHE pre-treatment group as compared to AHE administration after CP-intoxication. AHE pre-treatment preserved the enzyme status comparable to silymarin treated group while significant difference was recorded in comparison to control, indicating that full restoration of enzyme activity was not achieved with either of the treatments. The difference in various cardiac functions biomarkers between the AHE alone and control group was not statistically significant.

### Influence of AHE on cardiac antioxidant enzyme status

Cardioprotective effect of various AHE treatments against CP induced adverse effects was supported by increased myocardial antioxidant enzyme activity. Significant (*p* < 0.0001) lessening in the myocardial antioxidant enzymes viz.; SOD, POD, CAT and QR were observed in contrast to control group (Table 3). AHE oral doses prior to and after CP intoxication prevented the suppression of enzyme activity in contrast to the CP alone treated group. Interestingly, administration of AHE prior to CP resulted in a complete reversal of CP-induced increase in tissue level of POD, SOD and CAT to the control values, while QR level remained significantly altered as compared to control group. In AHE pre-treatment, enzyme activity of POD, SOD, CAT and QR was similar to silymarin treated group, however AHE post-treatment group showed noteworthy (*p* < 0.001) difference in activity level of CAT and QR, in contrast to both AHE pretreated and silymarin treated groups.

**Table 3:** Effect of cisplatin (CP) and different treatments of extract AHE on cardiac tissue antioxidant enzymes

Group	POD (U/min)	SOD (U/mg protein)	CAT (U/min)	QR (nM/min/mg protein)
Control	10.66 ± 0.381	1.146 ± 0.037	15.76 ± 0.117	137.5 ± 0.735
CP	5.930 ± 0.537^***^	0.742 ± 0.023^***^	9.49 ± 0.1576^***^	85.96 ± 0.358^***^
AHE alone	10.92 ± 0.531^+++^	1.145 ± 0.048^+++^	15.66 ± 0.196^+++^	137.7 ± 0.392^+++^
CP + AHE	7.99 ± 0.572^**,+^	0.992 ± 0.041+	11.89 ± 0.379^***,+++,###^	117.5 ± 0.926^***,+++,###^
AHE + CP	9.14 ± 0.081^++^	1.074 ± 0.049^+++^	14.84 ± 0.185^+++^	133.3 ± 0.481^*,+++^
CP + Sily	9.40 ± 0.231^++^	1.094 ± 0.049^+++^	14.73 ± 0.224^+++^	131.7 ± 1.200^**,+++^

Data are represented mean ± S.E.M (*n* = 6). Asterisks ^*, **, ***^ indicates *p* < 0.05, 0.001, 0.0001 vs. control; ^+, ++, +++^ indicates *p* < 0.05, 0.001, 0.0001 vs. CP alone; ^#, ##, ###^ indicates *p* < 0.05, 0.001, 0.0001 vs. AHE + CP. Non-significant difference (*p* > 0.05) was recorded between control and AHE alone treated group in all parameters (One way ANOVA followed by Tukey’s multiple comparison tests)

Ameliorating influence of AHE against CP-induced alteration in myocardial tissue glutathione status i.e. GSH, GR, GST, γ-GT and GPx is presented in Table 4. A marked (*p* < 0.0001) decline in the activity level of glutathione enzymes were observed in the cardiac tissue of CP treated rats in comparison to that of control group. Pre administration of AHE before CP intoxication resulted in more significant (*p* < 0.001) increase in GSH, GR, GST, γ-GT and GPx levels as compared to AHE post administration, indicative of the protective effect of AHE against CP induced declines. AHE pre-treatment exhibit protection against CP induced alterations in cardiac antioxidant enzymes corresponding to that of silymarin treated group. AHE when orally administered alone, showed non-significant change in the level of above mentioned antioxidant enzymes compared to control stipulating the nontoxic effect of selected dose of AHE.

**Table 4:** Effect of cisplatin (CP) and different treatments of AHE on cardiac enzymatic antioxidant levels and GSH profile

Group	GSH (μM/g tissue)	GR (nM/min/mg protein)	GST (nM/min/mg protein)	γ-GT (nM/min/mg Protein)	GPx (nM/min/mg Protein)
Control	20.55 ± 0.280	154.9 ± 0.958	148.6 ± 0.665	303.5 ± 0.811	122.4 ± 0.639
CP	12.41 ± 0.278^***^	111.8 ± 1.569^***^	110.9 ± 1.002^***^	100.6 ± 0.947^***^	66.77 ± 0.924^***^
AHE alone	20.66 ± 0.236^+++^	155.6 ± 0.439^+++^	148.4 ± 0.816^+++^	306.9 ± 0.401^+++^	122.9 ± 0.285^+++^
CP + AHE	14.34 ± 0.23***^,++,###^	121.6 ± 1.086^***,+++,###^	123.9 ± 1.110^***,+++,###^	189.3 ± 0.559^***,+++,###^	81.51 ± 1.659^***,+++,###^
AHE + CP	18.43 ± 0.415^**,###^	147.3 ± 0.948^**,+++^	141.7 ± 0.60^**,+++^	293.9 ± 0.602^**,+++^	111.7 ± 0.602^***,+++^
CP + Sily	18.33 ± 0.293^**,###^	146.0 ± 1.055^**,+++^	141.0 ± 0.899^**,+++^	294.7 ± 0.999^**,+++^	115.1 ± 1.00^**,+++^

Data are represented mean ± S.E.M (*n* = 6). Asterisks ^*, **, ***^ indicates *p* < 0.05, 0.001, 0.0001 vs. control; ^+, ++, +++^ indicates *p* < 0.05, 0.001, 0.0001 vs. CP alone; ^#, ##, ###^ indicates *p* < 0.05, 0.001, 0.0001 vs. AHE + CP. Non-significant difference (*p* > 0.05) was recorded between control and AHE alone treated group in all parameters (One way ANOVA followed by Tukey’s multiple comparison tests)

### Influence of AHE on cardiac protein content and oxidative stress markers

Table 5 represents the effect of AHE and CP, and AHE with CP on cardiac tissue protein and oxidative stress biomarkers specifically H_2_O_2_, NO and MDA. CP single dose resulted in a notable (*p* < 0.0001) decrease in cardiac tissue protein content and significant (*p* < 0.0001) augmentation in oxidative stress biomarkers as compared to the control group. Administration of AHE before and after a single dose of CP resulted in marked increase in cardiac tissue protein content while significant decrease in the level of cisplatin-mediated oxidative products viz.; H_2_O_2_, NO and MDA were recorded in cardiac tissues as compared to CP alone treated group. Treatment of animals before CP administration preserved the protein content relative to control values while oxidative stress biomarkers levels remains significantly different from control group. Post treatment of animals that received single *i.p*. dose of CP seems to be less effective in providing protection against CP mediated oxidative damage to cardiomyocytes in comparison to its prior administration, implicating preventive effect of AHE. Silymarin was used as a reference drug and AHE pre-treatment produced equal protection against CP induced alteration in cardiac tissue protein content and level of oxidative stress markers. AHE alone when administered orally throughout the study period showed no change in above mention parameters as compared to control group.

**Table 5:** Effect of cisplatin (CP) and different treatments of AHE on cardiac tissue protein, oxidative stress markers and lipid peroxidation

Group	Protein (μg/mg Tissue)	H_2_O_2_ (nM/min/mg Tissue)	Nitrite content (NO μM/ml)	MDA (nM/min/mg protein)
Control	1.638 ± 0.033	1.932 ± 0.015	42.59 ± 0.552	2.874 ± 0.180
CP	1.061 ± 0.075^***^	5.975 ± 0.025^***^	75.72 ± 0.707^***^	7.711 ± 0.264^***^
AHE alone	1.614 ± 0.015^+++^	1.911 ± 0.049^+++^	41.72 ± 0.650^+++^	2.859 ± 0.086^+++^
CP + AHE	1.419 ± 0.028^*,+++^	5.016 ± 0.013^***,+++^	62.63 ± 0.765^***,+++^	5.774 ± 0.285^***,+++,###^
AHE + CP	1.536 ± 0.032^+++^	2.794 ± 0.005^**,+++^	49.93 ± 0.419^**,+++^	3.219 ± 0.189^+++^
CP + Sily	1.564 ± 0.019^+++^	2.717 ± 0.006^**,+++^	50.60 ± 0.322^**,+++^	3.225 ± 0.112^+++^

Data are represented mean ± S.E.M (*n* = 6). Asterisks ^*, **, ***^ indicates *p* < 0.05, 0.001, 0.0001 vs. control; ^+, ++, +++^ indicates *p* < 0.05, 0.001, 0.0001 vs. CP alone; ^#, ##, ###^ indicates *p* < 0.05, 0.001, 0.0001 vs. AHE + CP. Non-significant difference (*p* > 0.05) was recorded between control and AHE alone treated group in all parameters (One way ANOVA followed by Tukey’s multiple comparison tests)

### Histopathological assessment of heart

**Fig. 2:**
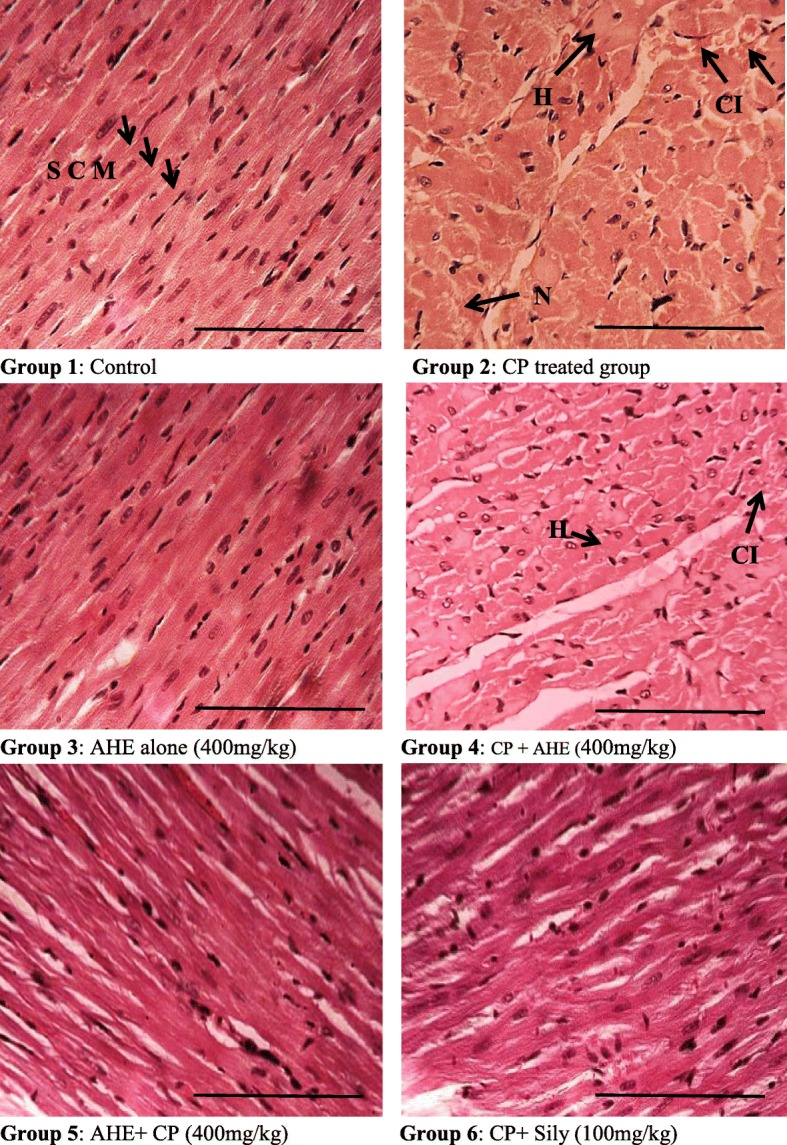
Histopathological effect of Cisplatin and protective effect of AHE in rat heart (H&E staining, magnification 40X). Group 1: Cardiac section from control rats showing normal morphology. Group 2: cardiac sections from CP-treated rats reveal degenerative changes. Group 3: Represents cardiac section from AHE alone treated rats. Group 4: AHE Post-treatment showed reduced degenerations. Group 5: AHE Pre-treatment results in significant protection against CP induced renal injury. Group 6: Showed protective effect of Silymarin treatment. AHE-*A.hydaspica* ethyl acetate fraction, CP-Cisplatin, SCM-striated cardiac muscles, H-hypertrophy, N-necrosis, CI-cellular infiltrations. Scale bar represent 100 μm

Histomicrographs of hematoxylin and eosin (H&E) stained transverse sections of the heart specimen from different treatment groups are shown in Fig. 2. The cardiac sections of distinct investigational groups indicated gradation of variations from no injury (control group and AHE alone treated group) to mild and medium lesions (AHE pre and post treated groups) to highly severe damages (CP alone group). Sections from the cardiac tissue of control and AHE alone treated rats represent the normal histoarchitecture of heart, normal typical cardiac muscle fibers with several small blood vessels, myofibrillar structure with striations, branched appearances and endurance with adjacent myofibrils and capillaries in the connective tissue and consistent acidophilic cytoplasm with central nucleus. Rats treated with CP alone showed massive degenerative changes, various grades of focal damages, hypertrophy of muscle fibers, distortion in blood capillaries, blood vessels were engorged with blood, disturbance in the trabeculae of heart and retrogressive lacerations in muscle fibres. Moreover, hyaline necrosis, leukocyte infiltration, mucoid edema and vacuolated muscle fibers are clearly visible in CP alone treated group. Remarkably, heart specimens from rats innoculated with CP and AHE displayed minimal deteriorating alterations, Pre-treatment of CP administered rats with either AHE or silymarin revealed regular cardiac muscle fibers with mild vicissitudes in restricted foci, less capillary dilatation and vacuolar alterations in contrast to CP-alone treated group, and maximum muscle fibers look as control group indicating protective effects of AHE pre-treatment. The protective influence of AHE pretreatment was comparable to silymarin group. AHE administration after CP intoxication displayed less recovery as compared to AHE pre-treatment group.

## Discussion

CP treatment initiates peroxidation of membrane bound polyunsaturated fatty acids and proteins lead to cardiomyocyte damage by impairing oxidant-antioxidant balance. It’s evident from previous studies that antioxidants are effective in cisplatin mediated toxicity [81]. Therefore, we aimed to check the influence *A. hydaspica* against CP induced myocardial insult. The protective effect of AHE against CP encouraged cardiac insult was evaluated by both pre and post treatment regimens. AHE was given to rats for 6 days after CP in AHE + CP and CP + AHE groups; in addition the AHE + CP group was exposed to AHE for 15 days before CP in order to check the preventive effect of pre-exposure. Silymarin was used as reference drug obtained from plant source. Previous research confirms that silymarin preclude lethal effects of anti-cancer drugs on heart.

Creatine kinase (CK), creatine kinase MB fraction (CK-MB), AST, ALT, LDH and cholesterol were measured as cardiac biomarker enzymes for estimation of cardiac insult and myocardial infarction [82, 83]. But above mentioned parameters are not circumscribed to cardiac functions apart from cTnI, CK and CK-MB, their enhanced amount in serum may possibly a depiction of non-cardiac tissue harms as well, for instance liver injury. cTnI and CK-MB are definitive indicator of myocardial impairment [84]. cTnI is one of the highly sensitive and specific parameters of myocardial damage. CP has the potential to disrupt the cell membranes, which enables the release of intracellular proteins such as cTnI, CK, and CK-MB. Previous studies also validated that CP treatment considerably increase plasma Troponin I activity, compared with control groups, following the administration of a single cisplatin dose [85]. We also observe significantly high cardiac cTnI, CK, and CK-MB levels in CP group. The heart injury could be a tributary event following irreversible alterations of cardiac cell membrane structure and function as a result of increased lipid peroxidation, which causes leakage of cardiac enzymes [20] and their concentration in the serum is marker of myocytes damage [86, 87]. CP induced toxicity was liable for enhancement of CK, CK-MB, LDH, cholesterol and cTnI concentration in the serum as compared to control. These outcomes are in covenant with the previous finding that CP-induced free radical production causes cardiac myocytes degeneration and membrane peroxidation, which increased the CK and CK-MB content of serum [20]. Our results coincides with the observations of EL-Sayed and colleagues signifying that in DOX treated groups CK-MB, CK and LDH were extremely elevated, indicating rigorously impaired cardiac tissue by DOX [88]. In addition ventricular changes, ongoing myocyte deterioration and curtailed coronary reserve might be the reasons of enzyme leakage [89]. Normalization of the serum content of CK, CK-MB, LDH and cholesterol in experimental groups treated with AHE shows enhanced cardiac function in CP intoxicated group, hence reveals the cardio-protective effect of *A. hydaspica*. Another investigation reported that pretreatment with silymarin ameliorated the cardiac marker enzymes and myocyte damage induced by isoproterenol compared with only isoproterenol-administered rats [90]. Silymarin treatment secures CP prompted myocardial injury by adjusting cardiac function biomarkers by augmenting endogenous antioxidants. Silymarin induced stabilization of cardiac membranes and stop leakage of enzymes [81]. These outcomes coincide with histological investigation.

GSH, GPx, SOD, POD, CAT, QR, γ GT and GST are endogenous antioxidant enzymes provides resistance against oxidative stress imposed tissue damage. Toxic agents induced decline in cellular GSH activity is linked directly with lipid peroxidation [91]. CP induced exhaustion of glutathione and allied antioxidants alters the cellular redox status and leads to the accumulation of endogenous ROS [92]. Rosic et al. 2016 revealed that cisplatin treatment intensify seepage of cardiac enzymes and lessen coronary flow escorted by augmented oxidative stress while *n*-acetyl cysteine (a precursor of glutathione) with antioxidant actions could diminish CP adverse effects. The current outcomes validate prior findings that myocardial glutathione and SOD levels were abridged in cardiac tissue apart from the higher level of oxidative stress in CP inoculated rats [20, 81]. Our results revealed that AHE + CP treatment antagonized the CP prompted reduction of antioxidant enzymes in similar manner to silymarin. The protection afforded by AHE may be mediated through the augmentation of cellular antioxidant enzymes and by precluding the generation of oxidative stress in myocardial cells.

Histopathological evaluation demonstrated myocardial atrophy, nuclear condensation of chromosomes and cytoplasmic vacuoles in the CP treated rat heart. Concerning the relative studies of CP with other toxic elements on cardiac tissue, same toxic influences were detected by adriamycin [93], doxorubicin [94] and isoproterenol [95]. Administration of AHE either before or after CP intoxication revealed substantial preclusion in the morphological vicissitudes. The observed effect might be due to the antioxidant potential of plant extract [28] and presence of bioactive metabolites (Table 1). Similarly Wang et al. illustrated that resveratrol exert protective effect against CP induced cardiotoxicity by alleviating the oxidative damage through its antioxidant potential [96]. CP prompted fatty impediment in the blood vessels and nuclear deterioration as well. All these morphological deteriorations caused by CP were noticeably returned by AHE + CP treatment. The observed protective effect may be endorsed to the distinct or mutual effect of active metabolites of AHE fraction.

## Conclusion

The consequence of oxidative stress in the mechanism of chemotherapeutic drug persuaded cardiac disorders intends that medicinal plants with antioxidant potential signifies a hopeful avenue for management. Plans for the management and preclusion of cardiovascular illnesses requires considerate of mechanism by which the prophylactic agents may possibly preclude the lethal effects. AHE may be advantageous for CP-prompted cardiotoxicity by hampering oxidative trauma. However, this warrants further investigations to confirm the mechanism of action and develop approaches against CP-persuaded cardiotoxicity.

## Data Availability

All the data is contained in the manuscript, raw datasets used and/or analysed during the current study is available from the corresponding author on reasonable request.

## Abbreviations

AHE: *Acacia hydaspica* ethyl acetate fraction
ALT: Alanine Aminotransferase
AST: Aspartate transaminase
CK: creatine kinase
CK-MB: Creatine kinase isoenzymes MB
CP: Cisplatin
cTnI: Cardiac Troponin I
H_2_O_2_: Hydrogen peroxide
LDH: Lactate dehydrogenase
No: Nitroc oxide

## References

[1] Al-Majed AA, Sayed-Ahmed MM, Al-Yahya AA, Aleisa AM, Al-Rejaie SS, Al-Shabanah OA (2006). Propionyl-L-carnitine prevents the progression of cisplatin-induced cardiomyopathy in a carnitine-depleted rat model. Pharmacol Res.

[2] Shanmugasundaram S, Bharathithasan R, Elangovan S (2002). 5-fluorouracil-induced cardiotoxicity. Indian Heart J.

[3] Chvetzoff G, Bonnotte B, Chauffert B (1998). Anticancer chemotherapy. Prevention of toxicity. Presse medicale.

[4] Pai VB, Nahata MC (2000). Cardiotoxicity of chemotherapeutic agents. Drug Saf.

[5] Razavi BM, Karimi G (2016). Protective effect of silymarin against chemical-induced cardiotoxicity. Iranian journal of basic medical sciences.

[6] Cheriparambil KM, Vasireddy H, Kuruvilla A, Gambarin B, Makan M, Saul BI (2000). Acute reversible cardiomyopathy and thromboembolism after Cisplatin and 5-fluorouracil chemotherapy a case report. Angiology.

[7] Weijl N, Wipkink-Bakker A, Lentjes E, Berger H, Cleton F, Osanto S (1998). Cisplatin combination chemotherapy induces a fall in plasma antioxidants of cancer patients. Ann Oncol.

[8] Wozniak K, Czechowska A, Blasiak J (2004). Cisplatin-evoked DNA fragmentation in normal and cancer cells and its modulation by free radical scavengers and the tyrosine kinase inhibitor STI571. Chem Biol Interact.

[9] Saleh RM, Awadin WF, Elseady YY, Waheish FE (2014). Renal and cardiovascular damage induced by cisplatin in rats. Life Sci J.

[10] Desoize B, Madoulet C (2002). Particular aspects of platinum compounds used at present in cancer treatment. Crit Rev Oncol Hematol.

[11] Lebwohl D, Canetta R (1998). Clinical development of platinum complexes in cancer therapy: an historical perspective and an update. Eur J Cancer.

[12] Ali BH, Al Moundhri MS (2006). Agents ameliorating or augmenting the nephrotoxicity of cisplatin and other platinum compounds: a review of some recent research. Food Chem Toxicol.

[13] Yagmurca M, Bas O, Mollaoglu H, Sahin O, Nacar A, Karaman O, Songur A (2007). Protective effects of erdosteine on doxorubicin-induced hepatotoxicity in rats. Arch Med Res.

[14] Yousef M, Saad A, El-Shennawy L (2009). Protective effect of grape seed proanthocyanidin extract against oxidative stress induced by cisplatin in rats. Food Chem Toxicol.

[15] HemaIswarya S, Doble M (2006). Potential synergism of natural products in the treatment of cancer. Phytother Res.

[16] Mansour HH, Hafez HF, Fahmy NM (2006). Silymarin modulates cisplatin-induced oxidative stress and hepatotoxicity in rats. J Biochem Mol Biol.

[17] Abdelmeguid NE, Chmaisse HN, Zeinab NA (2010). Protective effect of silymarin on cisplatin-induced nephrotoxicity in rats. Pak J Nutr.

[18] Borsari M, Gabbi C, Ghelfi F, Grandi R, Saladini M, Severi S, Borella F (2001). Silybin, a new iron-chelating agent. J Inorg Biochem.

[19] Valenzuela A, Aspillaga M, Vial S, Guerra R (1989). Selectivity of silymarin on the increase of the glutathione content in different tissues of the rat. Planta Med.

[20] El-Awady E-SE, Moustafa YM, Abo-Elmatty DM, Radwan A (2011). Cisplatin-induced cardiotoxicity: mechanisms and cardioprotective strategies. Eur J Pharmacol.

[21] Seigler DS (2003). Phytochemistry of Acacia—sensu lato. Biochem Syst Ecol.

[22] Sakthivel K, Kannan N, Angeline A, Guruvayoorappan C (2012). Anticancer activity of Acacia nilotica (L.) wild. Ex. Delile subsp. indica against Dalton’s ascitic lymphoma induced solid and ascitic tumor model. Asian Pac J Cancer Prev.

[23] Duarte MR, Wolf S (2005). Anatomical characters of the phyllode and stem of Acacia podalyriifolia A. Cunn. ex G. Don (Fabaceae). Rev Bras.

[24] Chakrabarty T, Gangopadhyay M (1996). The genus Acacia P. miller (Leguminosae: Mimosoideae) in India. J Econ Taxon Bot.

[25] Afsar T, Razak S, Khan MR, Mawash S, Almajwal A, Shabir M, Haq IU. Evaluation of antioxidant, anti-hemolytic and anticancer activity of various solvent extracts of Acacia hydaspica R. Parker aerial parts. BMC Complement Altern Med. 2016. 2016;16–28. 10.1186/s12906-016-1240-8 PMC4966721 27473625

[26] Afsar T, Khan MR, Razak S, Ullah S, Mirza B (2015). Antipyretic, anti-inflammatory and analgesic activity of Acacia hydaspica R. Parker and its phytochemical analysis. BMC Complement Altern Med.

[27] Afsar T, Razak S, Khan MR, Almajwal A (2017). Anti-depressant and anxiolytic potential of Acacia hydaspica R. Parker aerial parts extract: Modulation of brain antioxidant enzyme status. BMC Complement Altern Med.

[28] Afsar T, Razak S (2017). Modulatory influence of Acacia hydaspica R. Parker ethyl acetate extract against cisplatin inveigled hepatic injury and dyslipidemia in rats. BMC Complement Altern Med.

[29] Afsar T, Razak S, Almajwal A (2017). Acacia hydaspica ethyl acetate extract protects against cisplatin-induced DNA damage, oxidative stress and testicular injuries in adult male rats. BMC Cancer.

[30] Afsar T, Trembley JH, Salomon CE, Razak S, Khan MR, Ahmed K (2016). Growth inhibition and apoptosis in cancer cells induced by polyphenolic compounds of Acacia hydaspica: involvement of multiple signal transduction pathways. Sci Rep.

[31] Kozluca O, Olcay E, Sürücü S, Güran Z, Kulaksiz T, Üskent N (1996). Prevention of doxorubicin induced cardiotoxicity by catechin. Cancer Lett.

[32] Ojha Shreesh, Al Taee Hasan, Goyal Sameer, Mahajan Umesh B., Patil Chandrgouda R., Arya D. S., Rajesh Mohanraj (2016). Cardioprotective Potentials of Plant-Derived Small Molecules against Doxorubicin Associated Cardiotoxicity. Oxidative Medicine and Cellular Longevity.

[33] Elderbi MA, Mohamed A-WH, Hadi A-HA, Dabobash MD (2014). Potential protective effect of gum ARABIC against doxorubicin-induced CARDIOTOXICITY in WISTAR albino rats. Int J Pharm Sci Res.

[34] Afsar T, Razak S, Batoo KM, Khan MR (2017). Acacia hydaspica R. Parker prevents doxorubicin-induced cardiac injury by attenuation of oxidative stress and structural Cardiomyocyte alterations in rats. BMC Complement Altern Med.

[35] Turner P, Granville-Grossman K, Smart J (1965). Effect of adrenergic receptor blockade on the tachycardia of thyrotoxicosis and anxiety state. Lancet.

[36] Ojha Shreesh, Venkataraman Balaji, Kurdi Amani, Mahgoub Eglal, Sadek Bassem, Rajesh Mohanraj (2016). Plant-Derived Agents for Counteracting Cisplatin-Induced Nephrotoxicity. Oxidative Medicine and Cellular Longevity.

[37] Tikoo K, Bhatt DK, Gaikwad AB, Sharma V, Kabra DG (2007). Differential effects of tannic acid on cisplatin induced nephrotoxicity in rats. FEBS Lett.

[38] Ayodele JA, Ayodeji AO, Sunday AO, Scholarstical A, Oluwafemi TO (2015). Inhibitory effect of tannic acid and its derivative (gallic acid) against cisplatin–induced thiobarbituric acid reactive substances (TBARS) production in rat kidney–in vitro. Int J Adv Res.

[39] Ko H-H, Hung C-F, Wang J-P, Lin C-N (2008). Antiinflammatory triterpenoids and steroids from Ganoderma lucidum and G. tsugae. Phytochemistry.

[40] Beg S, Swain S, Hasan H, Barkat MA, Hussain MS (2011). Systematic review of herbals as potential anti-inflammatory agents: recent advances, current clinical status and future perspectives. Pharmacogn Rev.

[41] Salvador JA, Carvalho JF, Neves MA, Silvestre SM, Leitão AJ, Silva MM, Sá e Melo ML (2013). Anticancer steroids: linking natural and semi-synthetic compounds. Nat Prod Rep.

[42] Yasuhiro K, Jiro U, Toyofumi U, Tetsuo Y, Joichi K (1993). Prophylactic effect of methylprednisolone against cisplatin-induced nephrotoxicity in rats. Toxicol Lett.

[43] Lu Jin-Jian, Bao Jiao-Lin, Chen Xiu-Ping, Huang Min, Wang Yi-Tao (2012). Alkaloids Isolated from Natural Herbs as the Anticancer Agents. Evidence-Based Complementary and Alternative Medicine.

[44] Domitrović R, Cvijanović O, Pernjak-Pugel E, Škoda M, Mikelić L, Crnčević-Orlić Ž (2013). Berberine exerts nephroprotective effect against cisplatin-induced kidney damage through inhibition of oxidative/nitrosative stress, inflammation, autophagy and apoptosis. Food Chem Toxicol.

[45] Afsar T, Khan MR, Razak S, Ullah S, Mirza B (2015). Antipyretic, anti-inflammatory and analgesic activity of Acacia hydaspica R. Parker and its phytochemical analysis. BMC Complement Altern Med.

[46] Athira K, Madhana RM, Lahkar M (2016). Flavonoids, the emerging dietary supplement against cisplatin-induced nephrotoxicity. Chem Biol Interact.

[47] Egan D, O'kennedy R, Moran E, Cox D, Prosser E, Thornes RD (1990). The pharmacology, metabolism, analysis, and applications of coumarin and coumarin-related compounds. Drug Metab Rev.

[48] Al-Amiery AA, Al-Majedy YK, Kadhum AAH, Mohamad AB (2015). Novel macromolecules derived from coumarin: synthesis and antioxidant activity. Sci Rep.

[49] Nasr T, Bondock S, Youns M (2014). Anticancer activity of new coumarin substituted hydrazide–hydrazone derivatives. Eur J Med Chem.

[50] Sreedevi A, Bharathi K, Prasad K (2010). Protective effect of rutin against cisplatin-induced nephrotoxicity in rats. J Nat Remedies.

[51] Zhou Kequan, Raffoul Julian J. (2012). Potential Anticancer Properties of Grape Antioxidants. Journal of Oncology.

[52] Chen M, Meng H, Zhao Y, Chen F, Yu S (2015). Antioxidant and in vitro anticancer activities of phenolics isolated from sugar beet molasses. BMC Complement Altern Med.

[53] Kaur M, Agarwal C, Agarwal R (2009). Anticancer and cancer chemopreventive potential of grape seed extract and other grape-based products. J Nutr.

[54] Badhani B, Sharma N, Kakkar R (2015). Gallic acid: a versatile antioxidant with promising therapeutic and industrial applications. RSC Adv.

[55] Akomolafe SF, Akinyemi AJ, Anadozie SO. Phenolic acids (Gallic and tannic acids) modulate antioxidant status and Cisplatin induced nephrotoxicity in rats. Int Sch Res Notices. 2014;213–225. 10.1155/2014/984709 PMC4897306 27382634

[56] Evacuasiany E, Ratnawati H, Liana LK, Widowati W, Maesaroh M, Mozef T, Risdian C (2014). Cytotoxic and antioxidant activities of catechins in inhibiting the malignancy of breast cancer. Oxid Antioxid Med Sci.

[57] Shimizu M, Shirakami Y, Sakai H, Kubota M, Kochi T, Ideta T, Miyazaki T, Moriwaki H (2015). Chemopreventive potential of green tea catechins in hepatocellular carcinoma. Int J Mol Sci.

[58] Hassan SM, Khalaf MM, Sadek SA, Abo-Youssef AM (2017). Protective effects of apigenin and myricetin against cisplatin-induced nephrotoxicity in mice. Pharm Biol.

[59] Zhao C, Li C, Liu S, Yang L. The galloyl catechins contributing to main antioxidant capacity of tea made from Camellia sinensis in China. Sci World J. 2014;415–434. 10.1155/2014/863984 PMC4163330 25243234

[60] Malik S, Suchal K, Bhatia J, Gamad N, Dinda AK, Gupta YK, Arya DS (2016). Molecular mechanisms underlying attenuation of cisplatin-induced acute kidney injury by epicatechin gallate. Lab Invest.

[61] Sardana A, Kalra S, Khanna D, Balakumar P (2015). Nephroprotective effect of catechin on gentamicin-induced experimental nephrotoxicity. Clin Exp Nephrol.

[62] Kamatham S, Kumar N, Gudipalli P (2015). Isolation and characterization of gallic acid and methyl gallate from the seed coats of Givotia rottleriformis Griff. And their anti-proliferative effect on human epidermoid carcinoma A431 cells. Toxicol Rep.

[63] Hsieh T-J, Liu T-Z, Chia Y-C, Chern C-L, Lu F-J, Chuang M-c, Mau S-Y, Chen S-H, Syu Y-H, Chen C-H (2004). Protective effect of methyl gallate from Toona sinensis (Meliaceae) against hydrogen peroxide-induced oxidative stress and DNA damage in MDCK cells. Food Chem Toxicol.

[64] Guideline OO: 425: acute oral toxicity—up-and-down procedure. OECD Guidelines for the Testing of Chemicals 2001, 2:12–16.

[65] Irwin S (1968). Comprehensive observational assessment: Ia. A systematic, quantitative procedure for assessing the behavioral and physiologic state of the mouse. Psychopharmacology.

[66] Mensah A, Mireku E, Mensah M, Amponsah I (2014). Some neurological effects of the ethanolic stem bark extract of Cussonia bancoensis Aubrev and Pellgr (Araliaceae). J Pharmacogn Phytochemistry.

[67] Azu OO, Francis I, Abraham A, Crescie C, Stephen O, Abayomi O (2010). Protective agent, Kigelia Africana fruit extract, against Cisplatin-induced kidney oxidant injury in Sprague–Dawley rats. Asian J Pharma Clin Res.

[68] Charan J, Kantharia N (2013). How to calculate sample size in animal studies?. J Pharmacol Pharmacother.

[69] Nasr AY, Saleh HA (2014). Aged garlic extract protects against oxidative stress and renal changes in cisplatin-treated adult male rats. Cancer Cell Int.

[70] El-Halim SSA, Mohamed MM. Garlic Powder Attenuates Acrylamide-Induced Oxidative Damage in Multiple Organs in Rat. J Appl Sci Res. 2012;8(1):23–35.

[71] Lowry OH, Rosebrough NJ, Farr AL, Randall RJ (1951). Protein measurement with the Folin phenol reagent. J Biol Chem.

[72] Kakkar P, Das B, Viswanathan P (1984). A modified spectrophotometric assay of superoxide dismutase. Indian J Biochem Biophys.

[73] Jollow D, Mitchell J (1974). Zampaglione Na, Gillette J: Bromobenzene-induced liver necrosis. Protective role of glutathione and evidence for 3, 4-bromobenzene oxide as the hepatotoxic metabolite. Pharmacology.

[74] Habig WH, Pabst MJ, Jakoby WB (1974). Glutathione S-transferases the first enzymatic step in mercapturic acid formation. J Biol Chem.

[75] Carlberg I, Mannervik B (1975). Purification and characterization of the flavoenzyme glutathione reductase from rat liver. J Biol Chem.

[76] Mohandas J, Marshall JJ, Duggin GG, Horvath JS, Tiller DJ (1984). Differential distribution of glutathione and glutathione-related enzymes in rabbit kidney: possible implications in analgesic nephropathy. Biochem Pharmacol.

[77] Orlowski M, Sessa G, Green JP (1974). γ-Glutamyl transpeptidase in brain capillaries: possible site of a blood-brain barrier for amino acids. Science.

[78] Iqbal S, Bhanger M, Anwar F (2005). Antioxidant properties and components of some commercially available varieties of rice bran in Pakistan. Food Chem.

[79] Pick E, Mizel D (1981). Rapid microassays for the measurement of superoxide and hydrogen peroxide production by macrophages in culture using an automatic enzyme immunoassay reader. J Immunol Methods.

[80] Green LC, Wagner DA, Glogowski J, Skipper PL, Wishnok JS, Tannenbaum SR (1982). Analysis of nitrate, nitrite, and [15 N] nitrate in biological fluids. Anal Biochem.

[81] Abdelmeguid NE, Chmaisse HN, Abou Zeinab NS (2010). Silymarin ameliorates cisplatin-induced hepatotoxicity in rats: histopathological and ultrastructural studies. Pak J Biol Sci.

[82] Chrostek L (1988). Szmitkowski M: [enzymatic diagnosis of alcoholism-induced damage of internal organs]. Psychiatr Pol.

[83] Khan MR, Haroon J, Khan RA, Bokhari J, Rashid U (2011). Prevention of KBrO3-induced cardiotoxicity by Sonchus asper in rat. J Med Plants Res.

[84] Adams JE, Sicard GA, Allen BT, Bridwell KH, Lenke LG, Davila-Roman VG, Bodor GS, Ladenson JH, Jaffe AS (1994). Diagnosis of perioperative myocardial infarction with measurement of cardiac troponin I. N Engl J Med.

[85] Bertinchant J, Polge A, Juan J, Oliva-Lauraire M, Giuliani I, Marty-Double C, Burdy J, Fabbro-Peray P, Laprade M, Bali J (2003). Evaluation of cardiac troponin I and T levels as markers of myocardial damage in doxorubicin-induced cardiomyopathy rats, and their relationship with echocardiographic and histological findings. Clin Chim Acta.

[86] Kurian GA, Philip S, Varghese T (2005). Effect of aqueous extract of the Desmodium gangeticum DC root in the severity of myocardial infarction. J Ethnopharmacol.

[87] Rajadurai M, Prince PSM (2007). Preventive effect of naringin on cardiac markers, electrocardiographic patterns and lysosomal hydrolases in normal and isoproterenol-induced myocardial infarction in Wistar rats. Toxicology.

[88] El-Sayed EM, El-azeem ASA, Afify AA, Shabana MH, Ahmed HH (2011). Cardioprotective effects of Curcuma longa L. extracts against doxorubicin-induced cardiotoxicity in rats. J Med Plants Res.

[89] Potluri S, Ventura HO, Mulumudi M, Mehra MR (2004). Cardiac troponin levels in heart failure. Cardiol Rev.

[90] Rao PR, Viswanath RK (2007). Cardioprotective activity of silymarin in ischemia-reperfusion-induced myocardial infarction in albino rats. Exp Clin Cardiol.

[91] Kyle ME, Nakae D, Sakaida I, Serroni A, Farber JL (1989). Protein thiol depletion and the killing of cultured hepatocytes by hydrogen peroxide. Biochem Pharmacol.

[92] Arany Istvan, Safirstein Robert L (2003). Cisplatin nephrotoxicity. Seminars in Nephrology.

[93] Rajaprabhu D, Rajesh R, Jeyakumar R, Buddhan S, Ganesan B, Anandan R (2007). Protective effect of Picrorhiza kurroa on antioxidant defense status in adriamycin-induced cardiomyopathy in rats. J Med Plant Res.

[94] Singal PK, Iliskovic N (1998). Doxorubicin-induced cardiomyopathy. N Engl J Med.

[95] Upaganlawar A, Gandhi H, Balaraman R (2011). Isoproterenol induced myocardial infarction: protective role of natural products. J Pharmacol Toxicol.

[96] Wang J, He D, Zhang Q, Han Y, Jin S, Qi F (2009). Resveratrol protects against Cisplatin-induced cardiotoxicity by alleviating oxidative damage. Cancer Biother Radiopharm.

