# Correction to: Evaluating the protective potency of Acacia hydaspica R. Parker on histological and biochemical changes induced by Cisplatin in the cardiac tissue of rats

**DOI:** 10.1186/s12906-020-03117-w

**Published:** 2020-10-27

**Authors:** Tayyaba Afsar, Suhail Razak, Ali Almajwal, Maria Shabbir, Muhammad Rashid Khan

**Affiliations:** 1 Quaid I Azam Univ, Fac Biol Sci, Dept Biochem, Islamabad, Pakistan; 2 King Saud Univ, Dept Community Hlth Sci, Coll Appl Med Sci, Riyadh, Saudi Arabia; 3 NUST, Atta ur Rahman Sch Appl Biosci, Islamabad, Pakistan

**Correction to: BMC Complement Altern Med 19, 182 (2019)**

**https://doi.org/10.1186/s12906-019-2575-8**

Following publication of the original article [1], the authors identified an error in their affiliations. The correct affiliations should be as follows:

Tayyaba Afsar1,2*, Suhail Razak2*, Ali Almajwal2, Maria Shabbir1,3 and Muhammad Rashid Khan1

1 Quaid I Azam Univ, Fac Biol Sci, Dept Biochem, Islamabad, Pakistan. 2 King Saud Univ, Dept Community Hlth Sci, Coll Appl Med Sci, Riyadh, Saudi Arabia.3 NUST, Atta ur Rahman Sch Appl Biosci, Islamabad, Pakistan.
